# Supplementary material for: A personalized reinforcement learning recommendation algorithm using bi-clustering techniques
Source: PLoS One. 2025 Feb 20;20(2):e0315533. doi: 10.1371/journal.pone.0315533 (PMC11841880; doi:10.1371/journal.pone.0315533)
Supplement: Attachment — Submitted filename: Response To Reviewers.docx [file pone.0315533.s001.docx]

**Original Manuscript ID:** PONE-D-24-18432

**Original Article Title:** A Personalized Reinforcement Learning Recommendation Algorithm using Bi-Clustering Techniques

**To:** Editor-in-Chief PLOSE one

**Re:** Response to reviewers

Dear Editor,

Thank you for allowing a resubmission of our manuscript, with an opportunity to address the reviewers’ comments.

We are uploading (a) our point-by-point response to the comments (below) (response to reviewers), (b) an updated manuscript with yellow highlighting indicating changes, and (c) a clean updated manuscript

without highlights.

Best regards,

Muhammad Waqar et al.

**Reviewer #1:** 
Q#01: Abstract should be more concrete shedding more light on the novelties of your work.

**Answer:**

Thank you for the suggestion. I have revised the abstract to more clearly highlight the novel aspects of our work, emphasizing the unique contributions and findings of the study.

Q#02: the datasets used for experimentation should be described in the abstract.

**Answer:**

Thank you for the feedback. The dataset used for experimentation has now been explicitly mentioned in the abstract, as requested, to provide clarity on the data foundation of the work."

Q#03: In which domain this research can be used?

**Answer:**

Thank you for the comment. I have updated the abstract to clearly specify the domains in which the research findings can be applied, providing a clearer context for the potential impact of the study.

Q#04: Give motivation of this research also in abstract?

**Answer:**

Thank you for the suggestion. I have revised the abstract to include a clear motivation for the research, outlining the problem it addresses and the significance of the study in advancing the field

Q#05: At line 44,45 “Moreover, with increased size of data, these algorithms’ performance decreases gradually”. Any reference of this? Or it is your own statement?

**Answer:**

Thank you for the pointing out.. I have added Reference 6, which supports our statement regarding the decrease in performance with larger data sizes."

Q#06: At line 46, 47 give reference of 1 or 2 relevant works and drawbacks. Rather than starting In this work we are…..

**Answer:**

"Thank you for the suggestion. I have revised the section between lines 47-53 to incorporate references 5, 6, and 7, which provide relevant background and highlight the drawbacks discussed in this work, replacing the initial phrasing 'In this work we are...'

Q#07: Line 48, 49 “In this work we are combining the benefits of biclustering with power of RL to generate personalized
recommendations”. Should be placed in abstract for readers’ interest.

**Answer:**

Thank you for the suggestion. I have incorporated complementary nature of Biclustering and reinforcement learning into the abstract to better capture readers' interest."

Q#08: Why there is a need to predict rating in RL based Recommender Systems?

**Answer:**

Thank you for the comment. I have addressed this query by adding a response in lines 94-97, explaining the need to predict ratings in RL-based recommender systems and the rationale behind this approach."

Q#09: At line 58,59 Biclusters on a squared grid prove to be a cost effective solution while traversing through environment. More appropriately Change it to “can be a cost effective….”

**Answer:**

Thank you for the suggestion. I have revised the sentence to 'can be a cost-effective solution...' as recommended, to better reflect the intended meaning."

Q#10: Figure 2 methodology should be more appropriate depicting your core work and more specific. Not generalized.

**Answer:**

Thank you for the feedback. As per our understanding, Figure 2 has been designed to highlight the core aspects of our work based on methodology. We believe now it accurately represents the key concepts and do not feel a change at this stage.

Q#11: Literature review should be relevant and precise not very broad. Like knowledge based systems, Context aware systems, Social network based systems be removed.
**Answer:**

Thank you for the suggestion. The literature review has been revised to be more focused and precise, with the broader topics such as knowledge-based systems, context-aware systems, and social network-based systems removed as per your recommendation."

Q#12: A more conclusive statement needs to be added in conclusion and future work section.

**Answer:**

Thank you for the feedback. The conclusion and future work section has been revised to include a more conclusive statement, summarizing the key findings and outlining clear directions for future research."

Q#13: All figures quality needs to be improved.

**Answer:**

Thank you for the comment. The quality of Figures 4, 5, and 6 has been improved as requested, ensuring better clarity and visual presentation."

Q#14: Line 417, 429, 438, 449, 459. The discussion part of algorithms should be in Literature review

**Answer:**

Thank you for the suggestion. The discussion on the algorithms from lines 417, 429, 438, 449, and 459 has been moved to the literature review section, as recommended, to provide better context and alignment with the existing research."

Q#15: Line 1140 to Line 1146, make this paragraph more meaningful and specific. Don’t use generic terms.

**Answer:**

Thank you for the feedback. The paragraph between lines 1140 and 1146 has been revised to make it more meaningful and specific, removing generic terms to enhance clarity and focus."

**Reviewer #2:**
Q#1. Abstract should be more concrete highlighting what you have done not what others have done.

**Answer:**

Thank you for the suggestion. The abstract has been revised to focus more concretely on the contributions of our work, highlighting what we have done rather than what others have done.

Q#2. Notations are not identical. For example, in Eq. (2), it is mentioned that, A, represents a bicluster but at several places like in figure 1, B, is used to denote a bicluster.

**Answer:**

Thank you for pointing this out. The notations have been made consistent throughout the paper. Specifically, the bicluster notation has been corrected, with 'A' and 'B' now used consistently as per the intended definitions, including in Equation (2) and Figure 1."

Q#3. Several grammatical mistakes like Let assume should be Let’s assume.

**Answer:**

Thank you for highlighting this issue. Efforts have been made to correct all grammatical mistakes throughout the paper, including changes like 'Let assume' to 'Let’s assume,' ensuring improved clarity and accuracy.

Q#4. Literature review should be relevant and precise not very broad. Like knowledge based systems, Context aware systems, Social network based systems be removed.

**Answer:**

Thank you for the suggestion. The literature review has been revised to be more focused and precise, with the broader topics such as knowledge-based systems, context-aware systems, and social network-based systems removed as per your recommendation.

Q#5. In section IV, you mentioned Table 4 shows representations of various grid positions of generated biclusters after applying various biclustering algorithms. Instead of word various write names of biclustering algorithm for more clarity.

**Answer:**

Thank you for the suggestion. I have revised Section IV to replace the word 'various' with the specific names of the biclustering algorithms applied, providing greater clarity regarding the methods used."
Q#6. Name of ML-Latest-Small dataset is not consistent in the whole manuscript.

**Answer:**

Thank you for pointing this out. The name 'ML-Latest-Small' has been made consistent throughout the manuscript to avoid any confusion and ensure uniformity.

Q#7. Authors mentioned in section V that RLRS1 [53] used only one biclustering algorithm to generate Biclusters, But which one? Name it.

**Answer:**

Thank you for the comment. The specific biclustering algorithm used in RLRS1 [53] to generate biclusters has been added in line 768 for clarity."
Q#8. Also name algorithms that RLRS2 used to generate biclusters and which quality measure was used?

**Answer:**

Thank you for the suggestion. The algorithms used by RLRS2 to generate biclusters, as well as the quality measure applied, have been specified in line 775 for clarity."

Q#9. Figure 4(b), shows the steps taken by agent while exploring squared grid for ML-100K dataset, but authors mentioned it as figure 5(b).

**Answer:**

Thank you for pointing this out. The reference to Figure 4(b) has been corrected to Figure 5(b) as appropriate. Additionally, the section 'Learning of an agent' from lines 852 to 898 has been revised for clarity and accuracy."

Q#10. Name of Large Average Submatrix Biclustering Algorithm is confused at many places, in section III(B)(4) it is Large Average Value Biclustering Algorithm. In table 7 it is Least Average Submatrices. What is this? Are they same or different?

**Answer:**

Thank you for pointing out the inconsistency. The correct name of the algorithm is 'Large Average Submatrix Biclustering Algorithm,' and this term has now been used consistently throughout the entire document to avoid any confusion.

Q#11. Conclusion and future work section should be more elaborated.

**Answer:**

Thank you for the suggestion. The 'Conclusion and Future Work' section has been revised to provide a more detailed and comprehensive summary of the findings, as well as clearer directions for future research.
